# Supplementary material for: The phased pan-genome of tetraploid European potato
Source: Nature. 2025 Apr 16;642(8067):389–97. doi: 10.1038/s41586-025-08843-0 (PMC12158759; doi:10.1038/s41586-025-08843-0)
Supplement: Supplementary file 5 — Supplementary Figs. 3–14, 20, 21, 23–27 and 34–44. [file 41586_2025_8843_MOESM5_ESM.zip › suppl_figure_34-44/suppl_figure_37_D_v3.pdf]

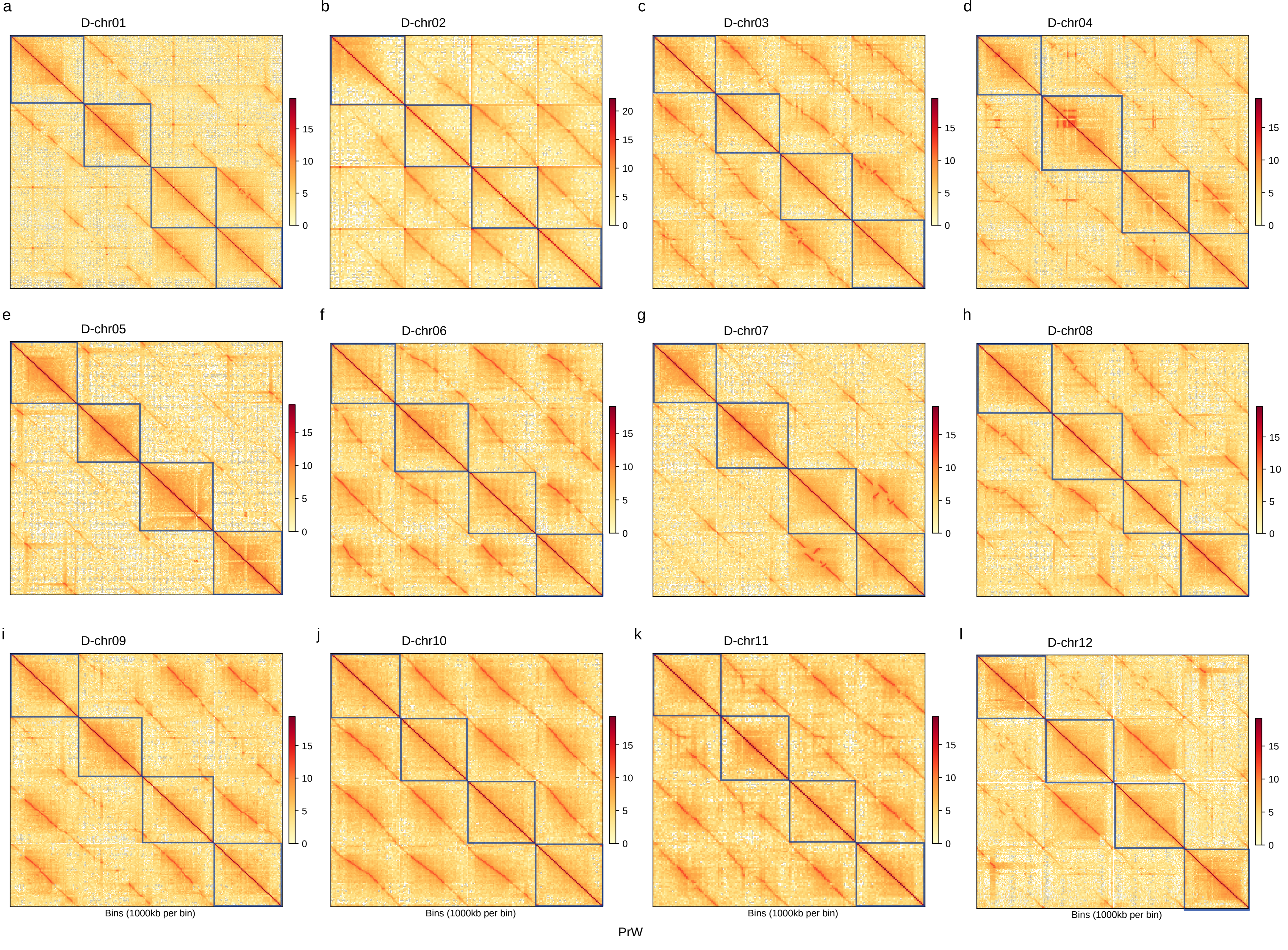
a
D-chr01
b
D-chr02
c
D-chr03
d
D-chr04
e
D-chr05
f
D-chr06
g
D-chr07
h
D-chr08
i
D-chr09
j
D-chr10
k
D-chr11
l
D-chr12
Bins (1000kb per bin)
Bins (1000kb per bin)
Bins (1000kb per bin)
Bins (1000kb per bin)
PrW
